# Primary immunodeficiencies associated with eosinophilia

**DOI:** 10.1186/s13223-016-0130-4

**Published:** 2016-05-24

**Authors:** Behdad Navabi, Julia Elizabeth Mainwaring Upton

**Affiliations:** Department of Paediatrics, The Hospital for Sick Children, University of Toronto, 555 University Avenue, Toronto, ON M5G-1X8 Canada; Division of Immunology and Allergy, Department of Paediatrics, The Hospital for Sick Children, University of Toronto, 555 University Avenue, Toronto, ON M5G-1X8 Canada

**Keywords:** Eosinophilia, Primary immunodeficiency disorders, Severe eosinophilia, Eosinophilia differentials

## Abstract

**Background:**

Eosinophilia is not an uncommon clinical finding. However, diagnosis of its cause can be a dilemma once common culprits, namely infection, allergy and reactive causes are excluded. Primary immunodeficiency disorders (PID) are among known differentials of eosinophilia. However, the list of PIDs typically reported with eosinophilia is small and the literature lacks an inclusive list of PIDs which have been reported with eosinophilia. This motivated us to review the literature for all PIDs which have been described to have elevated eosinophils as this may contribute to an earlier diagnosis of PID and further the understanding of eosinophilia.

**Methods:**

A retrospective PubMed, and Google Scholar search using the terms “eosinophilia” and “every individual PID” as classified by Expert Committee of the International Union of Immunological Societies with the limit of the English language was performed. Results were assessed to capture case(s) which reported eosinophilia in the context of PID conditions. Absolute eosinophil counts (AEC) were retrieved from manuscripts whenever reported.

**Results:**

In addition to the typical PID conditions described with eosinophilia, we document that MHC class II deficiency, CD3γ deficiency, STAT1 deficiency (AD form), Kostmann disease, cyclic neutropenia, TCRα deficiency, Papillon-Lefevre syndrome, CD40 deficiency, CD40L deficiency, anhidrotic ectodermal dysplasia with immune deficiency, ataxia-telangiectasia, common variable immunodeficiency disorders (CVID), Blau syndrome, CARD9 deficiency, neonatal onset multisystem inflammatory disease or chronic infantile neurologic cutaneous and articular syndrome (NOMID/CINCA), chronic granulomatous disease, MALT1 deficiency and Roifman syndrome have been noted to have elevated eosinophils. Severe eosinophilia (>5.0 × 10^9^/L) was reported in Omenn syndrome, Wiskott Aldrich syndrome, ADA deficiency, autoimmune lymphoproliferative syndrome, immunodysregulation polyendocrinopathy enteropathy X-linked, STAT3 deficiency, DOCK8 deficiency, CD40 deficiency, MHC II deficiency, Kostmann disease, Papillon-Lefevre syndrome, and CVID.

**Conclusions:**

This literature review shows that there is an extensive list of PIDs which have been reported with eosinophilia. This list helps clinicians to consider an extended differential diagnoses when tasked with exclusion of PID as a cause for eosinophilia.

**Electronic supplementary material:**

The online version of this article (doi:10.1186/s13223-016-0130-4) contains supplementary material, which is available to authorized users.

## Background

Eosinophils are primarily tissue-dwelling cells found in relatively low numbers within the circulation (less than 400/mm^3^) [1–3]. Eosinophilia can be secondary to varied conditions as recently reviewed by Curtis et al. [2], and can be classified based on absolute eosinophil count (AEC) as mild: 0.5–1.5 × 10^9^/L, moderate: 1.5–5.0 × 10^9^/L, or severe: >5.0 × 10^9^/L [4].

Primary immunodeficiency is a known differential diagnosis of eosinophilia that needs to be ruled out, particularly in pediatrics, when more common differentials such as infection, allergy and reactive causes are excluded [2, 5–7]. Eosinophilia in association with PIDs is well known; however, few PIDs are typically described in association with eosinophilia [5–9]. Most recent reviews of PIDs with eosinophilia include Wiskott Aldrich syndrome (WAS), hyper IgE syndromes (HIES), Omenn syndrome (OS), immunodysregulation polyendocrinopathy enteropathy X-linked (IPEX) syndrome, and Netherton’s syndrome. Moreover, ZAP70 deficiency, autoimmune lymphoproliferative syndrome (ALPS), selective IgA deficiency, and adenosine deaminase (ADA) deficiency have also appeared in differential diagnosis of eosinophilia.

Knowledge of the PIDs reported to have eosinophilia could assist clinicians assessing patients with eosinophilia when PID is considered. Given the current small list of PIDs reported with eosinophilia, we considered if a more comprehensive list could be generated by reviewing the literature. This review aimed to primarily capture PIDs reported with eosinophilia, and secondarily determine degree of eosinophilia where possible. Finally, some possible mechanisms driving eosinophilia in PID are highlighted.

## Methods

A review of the literature was undertaken to generate a list of PIDs reported with eosinophilia and to attempt to determine the degree of eosinophilia. PubMed and subsequently Google Scholar searches with English language filter were performed using the terms “eosinophilia” AND “every individual PID” as classified by Expert Committee of the International Union of Immunological Societies (IUIS) [9]. Abstracts of the PubMed results, and the title and the text of Google Scholar results were reviewed to find any case reports, case series or review articles, in which case eosinophilia and absolute eosinophil counts (AEC) were sought in the manuscript. Moreover, references of review articles and case series were assessed for any additional cases. This led to inclusion of *CHD7* and *CARD11* as genetic defects of OS [10, 11], and PGM3 deficiency [6, 12]. Lastly, Roifman syndrome [13, 14] and MALT1 deficiency [15] were included due to authors’ knowledge that they have been reported with eosinophilia.

As the primary goal was to capture the PIDs reported with eosinophilia we did not cite every article which describes eosinophilia. Once a condition was captured, we aimed to cite references with reported AEC but not necessarily all references which described eosinophilia for that condition. No minimum number of reports was required to be included in this review.

## Results

Here, PIDs reported in association with eosinophilia are briefly described. The cumulative range of eosinophilia in each condition and the retrieved AECs with their sources are respectively compiled in Table 1 and Additional file 1: Table S1. Online Mendelian Inheritance in Man (OMIM) numbers are provided in brackets after each condition.

**Table 1:** Primary immunodeficiency disorders associated with eosinophilia

PID	Genetic defect/subtype (s)	Functional defect	Inheritance	AEC range (× 10^9^/L)^b^
Combined immunodeficiencies				
ADA Deficiency	Late onset ADA	Elevated lymphotoxic metabolites	AR	0.8–4.7
ZAP70 deficiency^a^	ZAP70	Intracellular signaling abnormality	AR	9.5
CD3γ deficiency^a^	*CD3G*	T-cell receptor expression defect	AR	0.2–0.7
MHC II deficiency^a^	*RFXANK*	Impaired antigen presentation by APCs	AR	3–10
TCR α deficiency	*TRAC*	T-cell receptor generation	AR	0.08–2.5
MALT1 deficiency	*MALT1*	NF-kB activation failure	AR	Not specified
OS^a^	*RAG 1/2*	T-cell receptor generation abnormality	AR	0.1–21.8
	*IL7RA*	Defect in IL-7 receptor α chain	AR	6.49
	*IL*-*2RG*	Cytokine receptor signaling abnormality	AR	15.56
	22q11.2	DiGeorge syndrome	AD	1.36– >15
	*CHD7*	Chromatin organization defects	AR	1.3–4.1
	*LIG4*	DNA DSB repair defect	AR	2.12
	*ADA*	Elevated lymphotoxic metabolites	AR	0.85–1.73
	*RMRP*	Mitochondrial RNA processing defects	AR	Not specified
	*CARD11*	TCR/BCR induced NF-kB activation failure	AR	Not specified
	*ARTEMIS*	DNA repair defect	AR	Not specified
Combined immunodeficiencies with associated or syndromic features				
Ataxia-Telangiectasia	*ATM*	DNA break repair defect	AR	Not specified
WAS^a^	*WAS*	Actin cytoskeleton abnormality	AR	0–8.32
NS	*SPINK5*	Pro-Th2 and stratum corneum detachment	AR	Not specified
HIES	*STAT3* ^a^	Intracellular signaling abnormality	AD	0.029–54.81
	*Tyk2*	Cytokine signaling abnormality	AR	0.29–0.8
	*DOCK8* ^a^	Cytoskeletal organization defects	AR	0.245–37.88
Predominantly antibody deficiencies				
CVID	Unknown	Unknown	Variable	0.385–1.562
CD40L deficiency	*CD40L*	Defects in Ig isotype switching	XL	0.5–1.5
CD40 deficiency^a^	*CD40*	Defects in Ig isotype switching	AR	0.8–13.5
Selective IgA deficiency		Unknown		0.672
Diseases of immune dysregulation				
IPEX^a^	*FOXP3*	Dysfunction of regulatory Tcells	XL	0.236–8.423
ALPS^a^	*TNFRSF6*	Failure of apoptosis	AD	1.33–35.46
	Other^c^	Failure of apoptosis		Not specified
Congenital defects of phagocyte number or function or both				
Kostmann disease	*HAX1*	Control of apoptosis	AR	0.09–1.30
Cyclic neutropenia	*ELANE*	“Gain-of-function” in the neutrophil granule	AD	Not specified
STAT1 deficiency^a^	*STAT1*	IFN-γ signalling defect	AD	11.1
PLS	*FPR1*	Defective chemotaxis of PMNs	AR	0.96–2.156
CGD	*CYBB*	Neutrophil oxidative burst deficiency	XL	0.786
Defects of innate immunity				
EDA-ID	*NEMO*	Failure of NEMO-induced NF-κB activation	XL	1.45
CARD9 deficiency	*CARD9*	Selective defect in defense against fungal infection	AR	Not specified
Autoinflammatory disorders				
NOMID/CINCA	*CIAS1*	Defect in regulation of inflammation and apoptosis	AD	0.728–3.441
Blau syndrome	*NOD2*	NF-κB activation causing excess inflammatory cytokine	AD	Not specified
Not classified by IUIS				
PGM3 deficiency	*PGM3*	Possibly signalling defects	AR	0–3.6
Roifman syndrome	*RNU4ATAC*	Disrupted minor intron splicing	AR	Not specified

^a^Conditions with severe eosinophilia

^b^The absolute eosinophil count(s) with further details and source references in Additional file 1: Table S1

^c^ *TNFSF6* or *CASP8* or *CAS10*

### Combined immunodeficiencies

#### ADA deficiency (#102700)

ADA deficiency leads to accumulation of toxic deoxy-ATP within cells and immunodeficiency [16–18]. Late-onset ADA deficiency has been reported with eosinophilia [18–22].

#### CD3γ deficiency (#186740)

CD3γ deficiency, unlike CD3 δ, ε, and ζ deficiency, tends to present as combined immunodeficiency with variable onset [23, 24]. Autoimmunity, normal protein-specific antibody responses, high IgE, eosinophilia, and atopic eczema have been described in CD3γ deficiency [23–25]

#### ZAP70 deficiency (#269840)

ZAP70 is a central signalling molecule in thymic selection of the CD4 and CD8 lineages [26, 27]. Patients may present with an atopic dermatitis-like skin rash, eosinophilia and elevated IgE [28–30].

#### MHC class II deficiency (#209920)

MHC class II plays a pivotal role in CD4 T cell development and function [31, 32]. Reduced CD4+T cells, hypogammaglobulinemia, and an inability to mount immune responses are among the immunologic characteristics [32]. Eosinophilia has been reported in few cases [31, 33].

#### TCRα deficiency (#615387)

*TRAC* mutation interferes with a functional TCRαβ receptor [34–36]. Eosinophilia, frequently recurring infections, failure to thrive, autoimmunity, eczema, organomegaly, and elevated IgE have been reported [37].

#### MALT1 deficiency (#604860)

MALT1 is a regulator of NF-κB signalling. Normal to very low B cells, normal immunoglobulin with chronically elevated IgE are described [38, 39]. Eosinophilia was noted in this condition [15].

#### Omenn syndrome (OS; #603554)

OS is characterized by erythroderma, lymphadenopathy, eosinophilia, and profound immunodeficiency in infants [11, 40, 41]. Hepatosplenomegaly, hypogammaglobulinemia with elevated IgE are among other features [11, 42]. The OS genetic defects reported to be associated with eosinophilia include: *RAG1/RAG2*, *ARTEMIS*, *ADA*, *CHD7*, *RMRP* [43, 44], *LIG4*, *IL*-*2RG*, *IL7RA*, and *CARD11* [10]. Defects in *AK2* were not included here as the only OS due to *AK2* defect did not comment on eosinophilia [45]. DiGeorge syndrome has also been rarely reported to present with OS and eosinophilia [46, 47].

### Combined immunodeficiencies with associated or syndromic features

#### Wiskott–Aldrich syndrome (WAS; #301000)

WAS classically presents with the triad of thrombocytopenia, eczema and recurrent infections [48, 49]. It is associated with elevated IgE and IgA, inability to generate antibody against polysaccharide antigens, and eosinophilia [49–51]. A review of 154 patients found that 31 % had eosinophilia [50].

#### Ataxia-telangiectasia (#208900)

Ataxia-telangiectasia is characterized by progressive neurological abnormalities, radiosensitivity, and variable immunodeficiency [52]. Laboratory findings often include hypogammaglobulinemia, lymphopenia, and reversed CD4/CD8 ratio [52]; eosinophilia has also been reported in few cases [53, 54].

#### Hyper-IgE syndromes (HIES)

##### AD-HIES (Job’s syndrome; #147060)

AD-HIES is distinguished by its connective tissue, skeletal system, and dentition involvements in addition to recurrent infections, atopic dermatitis, elevated IgE, and eosinophilia [55]. STAT3 is the key to signal transduction of many cytokines, and memory B cells generation and mutations are causative [56–58]. Eosinophilia is noted in 80 % of AD-HIES patients [59]. A gain of function of STAT3 (p.Y640F) has recently been identified in lymphocytic variant of hypereosinophilic syndrome [60]. Therefore STAT3 appears to have an important role in eosinophil regulation.

##### DOCK8 deficiency (#243700)

DOCK8 deficiency accounts for the majority of AR-HIES [61–64] and is characterized by extensive cutaneous viral infections (herpes simplex, varicella zoster, human papillomavirus, and molluscum contagiosum), central nervous system (CNS) complications, elevated IgE, and eosinophilia [61, 65, 66].

##### Tyk2 deficiency (#611521)

To date there have been few reported cases of Tyk2 deficiency [67, 68]. Only the first patient had features of HIES including atopic dermatitis, eosinophilia, and high serum IgE levels [69]. However, mycobacterial and/or viral infections have been the most common phenotype among these patients [67, 68]. Eosinophilia is described in 2 of 8 total reported cases [68, 69].

#### Comel–Netherton syndrome (NS; #256500)

NS presents with atopic manifestations, an ichthyotic skin condition, and bamboo hair shaft defects due to *SPINK5* mutations [70, 71]. Elevated IgE and eosinophilia are frequently seen [6, 70–74]. Smith et al. showed eosinophilia in 7 out of 44 patients with NS [75].

### Predominantly antibody deficiencies

#### Common variable immunodeficiency disorders (CVID)

CVID is one of the more common immunodeficiencies with variable phenotypes mostly presents by recurrent infections, and low IgG and IgA and/or IgM [9, 76, 77]. There are few cases of CVID with eosinophilia [78–80]; however, it is difficult to determine prevalence of eosinophilia in CVID.

#### CD40 ligand (CD40L) deficiency (#300386)

CD40L deficiency characterized by recurrent infections, low IgG and IgA, and normal to increased IgM [81]. Eosinophilia has been described [82–84], and in one patient it was reported along with *Cryptosporidium parvum* and *Cryptococcus neoformans* infections [84].

#### CD40 deficiency (#109535)

CD40 deficiency is clinically indistinguishable from CD40L deficiency [85]. Eosinophilia has been described with C*ryptosporidium* infection [86, 87].

#### Selective IgA deficiency (#137100)

IgA deficiency is usually asymptomatic and characterized by a decreased or absent level of serum IgA with normal IgG and IgM [9, 88, 89]. It is considered on differential diagnoses of secondary eosinophilia [8, 90, 91].

### Diseases of immune dysregulation

#### Immunodysregulation polyendocrinopathy enteropathy X-linked (IPEX; #304790)

IPEX is a regulatory T (Treg) cell defect typically presents early in life [92, 93]. T cells are quantitatively normal with normal proliferative responses to mitogens and antigens; however, Treg cells are markedly reduced or absent [94–96]. High IgE and eosinophilia are frequently reported in IPEX patients [92, 97, 98].

#### ALPS-FAS (#601859)

ALPS-FAS is a disorder of lymphocyte homeostasis due to FAS receptor protein [99, 100]. Pathognomonic elevated double-negative T lymphocytes (TCRαβ^+^CD4^−^CD8^−^) [101–103], anemia, thrombocytopenia, and eosinophilia are among the laboratory findings [99, 100, 104, 105]. In a report of 68 patients with ALPS, 11 patients were found to have eosinophilia [104].

### Congenital defects of phagocyte number or function or both

#### SCN3 (Kostmann disease; #610738)

Kostmann disease typically presents with recurrent bacterial infections from early infancy, severe non-cyclic neutropenia, maturation arrest of myeloid differentiation, and compensatory monocytosis and eosinophilia [106–109]. Eosinophilia is considered to be a component of the classic presentation.

#### Cyclic neutropenia (#162800)

Cyclic neutropenia presents with recurrent fever, oral ulcers, recurrent oropharyngeal infections and periodic neutropenia [106]. In a review by Lang et al. eosinophilia was seen in 8.6 % of pediatric and 3.7 % of adult cases [110].

#### Papillon-Lefevre syndrome (PLS; #245000)

PLS is characterized by diffuse palmoplantar hyperkeratosis, rapid progressive periodontitis involving both deciduous and permanent dentition [111, 112]. Laboratory findings include decreased peripheral CD3 and CD4, defective burst test, as well as few cases of eosinophilia [111–113].

#### X-linked chronic granulomatous disease (CGD; #306400)

CGD is characterized by susceptibility to catalase-positive bacterial and fungal infections [114]. One CGD patient with mild eosinophilia despite taking prednisone every other day for eosinophilic colitis was reported [115]. There have also been reports of eosinophilia and eosinophilic inflammatory conditions in CGD patients including eosinophilic gastroenteritis and eosinophilic cystitis [115, 116].

#### STAT1 deficiency (AD form) (#600555)

Partial STAT1 deficiency can present as Mendelian Susceptibility to Mycobacterial Disease due to IFN-γ signaling defects [117–119]. There is one case with persistent leukocytosis and hypereosinophilia in a 2 month of age child who later was diagnosed as STAT1 deficiency [118].

### Defects of innate immunity

#### Anhidrotic ectodermal dysplasia-immune deficiency (EDA-ID; NEMO deficiency; #300248)

NEMO deficiency has been reported in various diseases including ectodermal dysplasia, incontinentia pigment, and severe life threatening pyogenic and mycobacterial infections [120–122]. Most patients have low serum IgG levels, with variable levels of other immunoglobulin isotypes [120, 123]; eosinophilia has also been reported [122–125].

#### CARD9 deficiency (#212050)

CARD9 is a cytosolic adaptor protein involved in differentiation of naïve T cells to TH17 [126]. Its defects are associated with recurrent *Candida* infections as well as susceptibility to deep dermatophytosis [126, 127]. Eosinophilia and elevated IgE level have been reported [127–129].

### Autoinflammatory disorders

#### Neonatal onset multisystem inflammatory disease (NOMID) or chronic infantile neurologic cutaneous and articular syndrome (NOMID/CINCA; #607115)

NOMID/CINCA is characterized by the triad of rash, CNS involvements and arthropathy. Leukocytosis, thrombocytosis and eosinophilia, as well as elevated acute-phase reactants are among the laboratory findings [130–132].

#### Blau syndrome (BS; #186580)

BS is a non-caseating granulomatous disease characterized by triad of uveitis, arthritis, and dermatitis of varying morphology [133, 134]. Hypercalcemia, hypercalciuria, elevated angiotensin converting enzyme level, leukopenia, and eosinophilia are described laboratory findings [135–139].

### Not classified by IUIS

#### PGM3-deficiency

PGM3 deficiency presents with hyper-IgE–like features, atopy, autoimmunity and neurocognitive impairment [6, 12, 67, 140–142]. PGM3 is involved in posttranslational alterations necessary for functioning of many proteins and lipids [141]. Eosinophilia, inverted CD4/CD8 ratio, and increased IgE level were described [142].

#### Roifman syndrome

Roifman syndrome is characterized by bone dysplasia, growth retardation, retinal dystrophy and humoral immunodeficiency [13, 14]. In the original description of Roifman syndrome 3/4 patients had eosinophilia [13].

## Discussion

### Evaluation of possible PID in a patient with eosinophilia

Many patients with eosinophilia will be explained by secondary causes such as parasitic infections, allergies, or hematological problems which are well reviewed elsewhere [2, 5–7]. If PID is being considered as a potential cause of eosinophilia, a wide range of PIDs have been associated with eosinophilia including disorders of Tcell development and signalling, cytokine signalling, cytoskeletal formation, autoimmunity, thymic development, innate immunity, humoral immunity and phagocytic function.

The history and physical examination may reveal clues which lead to likely diagnoses and further immune evaluation. Despite lack of sensitive and or specific signs and symptoms in respect to PIDs, many red flags including specific patterns of infections, autoimmunity, need for intravenous antibiotics, and prolonged oral antibiotics use with little effects have been previously reviewed [143–145]. Additionally, a detailed practice parameter for the evaluation of PID was recently published [146]. A patient history including infections, autoimmunity, malignancy and a review of systems including the presence of constitutional symptoms, allergies, and diarrhea may assist in deciding the likelihood of an immunodeficiency. A family history including consanguinity, early deaths and malignancy will also assist in evaluating for serious causes. The physical exam may note growth parameters, dysmorphism, skin abnormalities, thrush, lymphatic tissue, skin/nail abnormalities and neurological features.

**Fig. 1:**
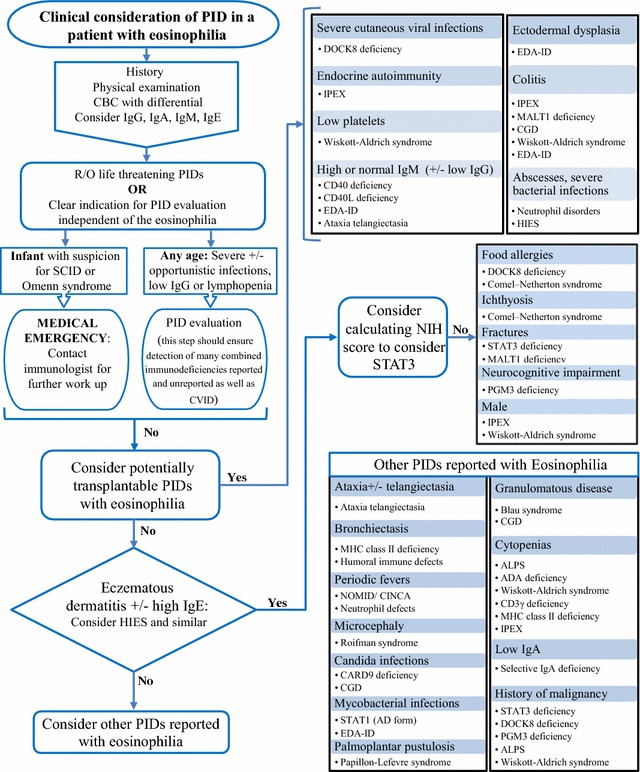
A Severity-Based Approach to Assessing for PIDs which have been reported with Eosinophilia. The initial approach presented here is based on the history and physical exam and simple laboratory tests. This assessment may reveal independent indications for PID evaluation independent of eosinophilia. In an infant we suggest a low threshold to consider SCID and Omenn syndrome. A consideration of transplantable/severe PIDs is presented next with some phenotypic clues. Then HIES and similarly presenting conditions may be considered in patients with eczematous dermatitis ± high IgE including performing the NIH score for STAT3 deficiency. Finally, phenotypic clues for other PIDs which have been reported with eosinophilia are listed. This approach does not suggest to evaluate all patients for all disorders but to begin with considering severe causes and then let the phenotype guide investigations for particular conditions. A few conditions, such as WAS and STAT3, appear in multiple locations due to variable presentations

A phenotypic guide to immunological conditions with eosinophilia has been published [6] which contains many of the conditions in this review. Here we present a complimentary approach which focuses on the severity of the conditions followed by some diagnostic clues (Fig. 1). After a detailed history and physical exam, quantitative immunoglobulins could be ordered if a clinical concern of PID exists. Laboratory results of a complete blood count (CBC) and differential will already be available if the reason for referral is eosinophilia. If the patient is lymphopenic or hypogammaglobulinemic then a work up for PID independent of the eosinophilia and comprehensive resources are recommended [146, 147]. A patient with SCID/Omenn syndrome presents a medical emergency so active consideration of these life threatening conditions is warranted in an infant. After considering SCID/Omenn syndrome, other significant or transplantable conditions could be considered such as WAS, IPEX, DOCK8 deficiency, EDA-ID and CD40L/CD40 deficiency and others. Next, consideration of the hyper IgE syndromes is suggested because they are well known to be associated with eosinophilia and some can be severe. Finally, a consideration of the other reported causes may be needed depending on the circumstances and whether an alternate diagnosis has been achieved.

## Limitations of this study

By the nature of a review of published literature, we are limited by what authors have reported. There may be some PIDs with eosinophilia which were not captured due to reporting omission. This limitation is not a weakness because the purpose was to see the basis of the assertion that PID should be considered in a patient with eosinophilia and to catalogue the previously reported conditions. Our strategy did allow the capture of even single case reports and documented many PIDs not typically thought to have been noted with eosinophilia but may have missed some diseases including those which may list the laboratory values in a table in a way not captured by our search, or in non-English language journals.

Determining the true frequency of eosinophilia in individual PID conditions is also subject to reporting omissions and biases. We have provided some information about how commonly eosinophilia has been noted such as from case series of patients, but we cannot provide an exact frequency with this methodology.

The AEC is not described for every PID and therefore the degree and the range of eosinophilia is derived from a low number of cases. The AEC range reported in Table 1 is the cumulative results of the cases which mentioned eosinophil count(s). They are intended to serve as a guide when considering severe eosinophilia. The degree of eosinophilia can be markedly varied in each PID. As summarized in Table 1 and reported in detail in Additional file 1: Table S1, there is a broad variability in the degree of eosinophilia associated with each individual PID and or subtype(s). Given the variability of the degree of eosinophilia this is unlikely to be of major diagnostic assistance, but severe eosinophilia is less common and may have more diagnostic utility.

## Conclusions

There are more PIDs documented with eosinophilia than typically recognized. Eosinophilia has been reported with many primary immunodeficiencies including severe combined, combined, humoral, phagocytic and innate immunodeficiencies. Based on the AEC derived from the literature:

- Severe eosinophilia was seen in OS, WAS, ALPS, STAT3 deficiency, DOCK8 deficiency, IPEX, CD40 deficiency, ZAP70 deficiency, STAT1 deficiency (AD form) and MHC class II deficiency.
- Lesser degree of eosinophilia was described in ADA deficiency, CD3γ deficiency, TCRα deficiency, MALT1 deficiency, Ataxia-telangiectasia, PGM3 deficiency, Tyk2 deficiency, NS, CVID, IgA deficiency, CD40L deficiency, SCN3, cyclic neutropenia, PLS, CGD, EDA-ID, CARD9 deficiency, NOMID/CINCA, Blau syndrome, and Roifman syndrome.

We agree with previous reviews that PIDs should be considered in patients with eosinophilia, especially children, when typical causes have been ruled out [2, 5–8]. This list of reported PID conditions with eosinophilia will help with the assessment of such patients. Eosinophilia can be driven by varied processes including imbalances in Th1/Th2, cytokine derangements, infections and medications.

## Additional file

10.1186/s13223-016-0130-4 Absolute eosinophil counts (AEC) in Primary Immunodeficiency Diseases (PID): number of patients reported and references cited.

## Abbreviations

PID: primary immunodeficiency disorder
WAS: Wiskott Aldrich syndrome
AEC: absolute eosinophil count
IPEX: immunodysregulation polyendocrinopathy enteropathy X-linked
OS: Omenn syndrome
ADA: adenosine deaminase
IUIS: International Union of Immunological Societies
ALPS: autoimmune lymphoproliferative syndrome
TCR: T cell receptor
HIES: hyper-IgE syndromes
NS: Comel–Netherton syndrome
CGD: X-linked chronic granulomatous disease
EDA-ID: anhidrotic ectodermal dysplasia-immune deficiency
PLS: Papillon-Lefevre syndrome
CNS: central nervous system
CVID: common variable immunodeficiency disorders
NOMID/CINCA: neonatal onset multisystem inflammatory disease or chronic infantile neurologic cutaneous and articular syndrome
CBC: complete blood count
