# Supplementary material for: Primary immunodeficiencies associated with eosinophilia
Source: Allergy Asthma Clin Immunol. 2016 May 24;12:27. doi: 10.1186/s13223-016-0130-4 (PMC4878059; doi:10.1186/s13223-016-0130-4)
Supplement: Supplementary file 1 — 10.1186/s13223-016-0130-4 Absolute eosinophil counts (AEC) in Primary Immunodeficiency Diseases (PID): number of patients reported and references cited. [file 13223_2016_130_MOESM1_ESM.docx]

**Supplementary Table S1*.** Absolute eosinophil counts (AEC) in Primary Immunodeficiency Diseases (PID): number of patients reported and references cited.

| **PID** | **# of**  **Patients** | **Genetic Defect** | **AEC**  **(x 10^9^/L)** | **Ref** | **PID** | **# of**  **Patients** | **Genetic Defect** | **AEC**  **(x 10^9^/L)** | **Ref** |
| --- | --- | --- | --- | --- | --- | --- | --- | --- | --- |
| ADA def | 1 | *ADA* | 1-2.8 | [[18](#_ENREF_18)] | HIES | 9 | *STAT3* | 0.7-10.045 | [[148](#_ENREF_149)] |
|  | 1 | *ADA* | 0.8-4.7 | [[21](#_ENREF_21)] |  | 4 | *STAT3* | 0.029-0.094 | [[149](#_ENREF_150)] |
| ZAP70 def | 1 | ZAP70 | 9.5 | [[29](#_ENREF_29)] |  | 1 | *STAT3* | 54.81 | [[150](#_ENREF_151)] |
| TCR α def | 2 | *TRAC* | 0.08-2.5 | [[37](#_ENREF_37)] |  | 1 | *STAT3* | 1.608 | [[151](#_ENREF_152)] |
| OS | 1 | ND** | >5 | [[152](#_ENREF_153)] |  | 1 | *STAT3* | 1.0215 | [[153](#_ENREF_154)] |
|  | 1 | ND** | 12.47 | [[154](#_ENREF_155)] |  | 1 | *STAT3* | 1.3 | [[155](#_ENREF_156)] |
|  | 9 | *RAG1* (2)  ND** (7) | 0.03-12.1 | [[42](#_ENREF_42)] |  | 13 | AR- HIES | 2.61-17.874 | [[65](#_ENREF_65)] |
|  |  |  |  |  |  | 21 | *DOCK8* | 0.29-37.88 | [[61](#_ENREF_61)] |
|  | 3 | *RAG 1* | 1.4-5.2 | [[156](#_ENREF_157)] |  | 11 | *DOCK8* | <0.6 – 33.0 | [[62](#_ENREF_62)] |
|  | 3 | *RAG1* | 0.1-15.1 | [[157](#_ENREF_158)] |  | 64 | *DOCK8* | 0.245-37.88 | [[158](#_ENREF_159)] |
|  | 1 | *RAG1* | 7.1 | [[159](#_ENREF_160)] |  | 1 | *DOCK8* | 36.0 | [[160](#_ENREF_161)] |
|  | 1 | *RAG1* | 1.3 | [[161](#_ENREF_162)] |  | 1 | *TyK2* | 0.7-0.8 | [[69](#_ENREF_69)] |
|  | 1 | *RAG1* | 21.8 | [[162](#_ENREF_163)] |  | 1 | *TyK2* | 0.29 | [[163](#_ENREF_164)] |
|  | 1 | *RAG1* | 21.8 | [[164](#_ENREF_165)] | PGM3 def | 9 | *PGM3* | 0.7-2.0 | [[142](#_ENREF_142)] |
|  | 9 | *RAG1* in 7  *RAG2* in 2 | 0.5-10.7 | [[165](#_ENREF_166)] |  | 8 | *PGM3* | 0.0-3.6 | [[12](#_ENREF_12)] |
|  |  |  |  |  | CD40L def | 1 | *CD40L* | 0.5-1.5 | [[82](#_ENREF_82)] |
|  | 8 | *RAG2* (6);  ND** (2) | 2.8-8.1 | [[166](#_ENREF_167)] |  | 1 | *CD40L* | 0.88 | [[167](#_ENREF_168)] |
|  |  |  |  |  | CD40 def | 1 | *CD40* | > 5 | [[86](#_ENREF_86)] |
|  | 2 | *ADA* | 0.85-1.73 | [[17](#_ENREF_17)] |  | 2 | *CD40* | 0.8-13.5 | [[87](#_ENREF_87)] |
|  | 1 | *ARTEMIS* | 7.488 | [[168](#_ENREF_169)] | IPEX | 1 | *FOXP3* | 6.12 | [[92](#_ENREF_92)] |
|  | 2 | *CHD7* | 1.3-4.1 | [[169](#_ENREF_170)] |  | 14 | *FOXP3* | 0.498-8.423 | [[96](#_ENREF_96)] |
|  | 1 | *LIG4* | 2.12 | [[170](#_ENREF_171)] |  | 1 | *FOXP3* | 3.170 | [[171](#_ENREF_172)] |
|  | 1 | *IL7RA* | 6.49 | [[172](#_ENREF_173)] |  | 4 | *FOXP3* | 0.236-0.9 | [[173](#_ENREF_174)] |
|  | 1 | *IL2RG* | 15.56 | [[174](#_ENREF_175)] |  | 1 | *FOXP3* | 5.4 | [[175](#_ENREF_176)] |
|  | 5 | 22q11.2 | Max >15 | [[46](#_ENREF_46)] |  | 10 | *FOXP3* | 0.79-6 | [[176](#_ENREF_177)] |
|  | 2 | 22q11.2 | 1.36-5.22 | [[47](#_ENREF_47)] | ALPS | 1 | *TNFRSF6* | 2.89 | [[102](#_ENREF_102)] |
| WAS | 2 | *WAS* | 1.33-2.78 | [[51](#_ENREF_51)] |  | 11 | *TNFRSF6* (8);  ND** (3); | 1.33-35.46 | [[104](#_ENREF_104)] |
|  | 15 | *WAS* | 0-8.32 | [[177](#_ENREF_178)] |  |  |  |  |  |
|  | 1 | *WAS* | 0.742 | [[167](#_ENREF_168)] |  | 1 | *TNFRSF6* | 35.1 | [[178](#_ENREF_179)] |
| NOMID/  CINCA | 2 | *CIAS1* | 0.728-1.32 | [[179](#_ENREF_180)] | CD3γ def. | 5 | *CD3G* | 0.2-0.7 | [[23](#_ENREF_23)] |
|  | 1 | *CIAS1* | 3.441 | [[180](#_ENREF_181)] | SCN3 | 2 | ND | 0.285-0.6 | [[181](#_ENREF_182)] |
| PLS | 1 | *CTSC* | 2.156 | [[111](#_ENREF_111)] |  | 17 | *HAX1 (3);* ND** (14) | 0.09-1.30 | [[182](#_ENREF_183)] |
|  | 2 | ND** | 0.96-1.55 | [[113](#_ENREF_113)] |  |  |  |  |  |
| CVID | 1 | ND** | 1.425-1.562 | [[78](#_ENREF_78)] | IgA Def | 1 |  | 0.672 | [[183](#_ENREF_184)] |
|  | 1 | ND** | 0.385 | [[79](#_ENREF_79)] | CGD | 1 | gp91^phox^ | 0.786 | [[115](#_ENREF_115)] |
| EDA-ID | 1 | *NEMO* | 1.45 | [[124](#_ENREF_124)] | MHC II def | 4 | *RFXANK* | 3-10 | [[31](#_ENREF_31)] |
| STAT1 def | 1 | *STAT1* | 11.1 | [[118](#_ENREF_118)] |  | 1 | ND** | 0.5 | [[33](#_ENREF_33)] |

* Note that this table includes only the conditions with absolute eosinophil count(s) reported in the literature; ** Not Determined.
